# Screening for non-classic congenital adrenal hyperplasia in women: New insights using different immunoassays

**DOI:** 10.3389/fendo.2022.1048663

**Published:** 2023-01-10

**Authors:** Afif Nakhleh, Leonard Saiegh, Naim Shehadeh, Naomi Weintrob, Mohammad Sheikh-Ahmad, Lia Supino-Rosin, Sandra Alboim, Raya Gendelman, Moshe Zloczower

**Affiliations:** ^1^ Institute of Endocrinology, Diabetes and Metabolism, Rambam Health Care Campus, Haifa, Israel; ^2^ Diabetes and Endocrinology Clinic, Maccabi Healthcare Services, Haifa, Israel; ^3^ Ruth & Bruce Rappaport Faculty of Medicine, Technion, Israel Institute of Technology, Haifa, Israel; ^4^ Department of Endocrinology, Bnai Zion Medical Center, Haifa, Israel; ^5^ Department of Pediatrics, Sackler Faculty of Medicine, Tel Aviv University, Tel-Aviv, Israel; ^6^ Pediatric Endocrinology and Diabetes Unit, Dana-Dwek Children’s Hospital, Tel-Aviv Medical Center, Tel-Aviv, Israel; ^7^ Central Laboratory, Maccabi Healthcare Services, Rehovot, Israel; ^8^ The Endocrine Laboratory, Rambam Health Care Campus, Haifa, Israel

**Keywords:** non-classic congenital adrenal hyperplasia, radioimmunoassay, enzyme-linked immunosorbent assay, 17-hydroxyprogesterone, cosyntropin stimulation test, LH : FSH ratio

## Abstract

**Context:**

The 250µg-cosyntropin stimulation test (CST) is used to diagnose non-classic congenital adrenal hyperplasia (NCCAH). The current recommendation is to perform CST when follicular 17-hydroxyprogesterone (17OHP) is 6-30 nmol/L, a cutoff derived from radioimmunoassay (RIA). Recently, enzyme-linked immunosorbent assay (ELISA) has replaced RIA.

**Objectives:**

We aimed to (1) determine the RIA and ELISA-based 17OHP cutoffs at which CST should be performed, (2) identify predictors of NCCAH.

**Methods:**

A retrospective study at an Israeli Health Maintenance Organization. Data were retrieved from women with suspected NCCAH, referred for CST during 2001–2020. NCCAH was defined as a stimulated 17OHP >30 nmol/L. Serum 17OHP levels were assayed by RIA from 1/2000-3/2015, and by ELISA from 4/2015-12/2020. ROC curves were generated and optimal 17OHP thresholds were determined. Multivariate analysis was performed.

**Results:**

CST was performed in 2409 women (1564 in RIA, 845 in ELISA). NCCAH was diagnosed in 4.7% of the RIA group and 7.5% of the ELISA group. The optimal basal 17OHP cutoff values predicting NCCAH were 6.1 nmol/L in RIA (sensitivity=93.2%, specificity=91.7%) and 8.2 nmol/L in ELISA (sensitivity=93.7%, specificity=92.3%). In multivariate analysis, higher basal 17OHP, lower LH: FSH ratio, and oligomenorrhea were predictors of NCCAH in RIA. Higher basal 17OHP, androstenedione, and total testosterone were predictors of NCCAH in ELISA. A lower LH: FSH ratio showed similar trend in ELISA.

**Conclusions:**

Optimal RIA-based basal 17OHP cutoff was comparable with that recommended in guidelines. The results suggest adopting a higher 17OHP cutoff when using ELISA. LH : FSH ratio improves the negative predictive value of basal 17OHP.

## 1 Introduction

The 250 µg cosyntropin stimulation test (CST) is used to diagnose non-classic congenital adrenal hyperplasia (NCCAH) due to 21-hydroxylase deficiency. The current recommendation is to perform CST when basal serum 17-hydroxyprogesterone (17OHP) levels (performed in the early follicular phase in females) are 6-30 nmol/L, and the test is considered positive for NCCAH diagnosis when the 60-minutes post-CST 17OHP level is > 30 nmol/L (1). These 17OHP cutoffs were mainly derived from radioimmunoassay (RIA) data (2). The Endocrine Society recommends screening with an early morning (before 8 AM) basal serum 17OHP measurement by liquid chromatography with tandem mass spectrometry (LC-MS/MS) (1). However, because of the limited availability of LC-MS/MS, immunoassays remain the assays most frequently used.

There are several disadvantages to using RIA including reagent instability, radioactive waste management, and the need for manual handling. Recently, a validated enzyme-linked immunosorbent assay (ELISA) has widely replaced RIA in the measurement of serum 17OHP (3). ELISA is simple, easy to perform, and uses commercially available reagents (4). A validation study by an Australian laboratory showed that IBL’s 17OHP ELISA assay provides an acceptable alternative for the Siemens Healthcare Diagnostics 17OHP RIA (3, 5).

The 17OHP cutoff of 6 nmol/L for NCCAH screening has been questioned by several studies utilizing RIA assays that suggested lower cutoffs (6–9). Several studies showed that between 2% and 11% of adult patients with NCCAH might be missed using this approach (10). Implementation of a new assay justifies re-evaluation of the basal 17OHP threshold level for performing CST, especially in the era of cost-effective medicine.

Since most of the patients diagnosed with NCCAH are females (11, 12), we studied only females aged >16 years with clinical suspicion of NCCAH. The current study aimed to determine the best cutoff for performing CST using ELISA compared to RIA. In addition, we aimed to identify clinical and laboratory factors that could predict the diagnosis of NCCAH.

## 2 Materials and methods

This non-interventional, retrospective, cohort study was conducted using the electronic medical database of Maccabi Healthcare Services (MHS), a large health maintenance organization (HMO) in Israel serving over 2 million patients. All medical data were obtained from the MHS automated database. The retrieval of patients’ records was performed using MDClone, a query tool that provides comprehensive patient-level data for a wide range of variables in a defined time frame around an index event (13, 14). This platform was used to elicit information on demographic, clinical, and biochemical data, and dispensed community prescriptions. Approval was obtained from MHS institutional review board (IRB) and ethics committee to access and analyze data. Individual patient informed consent was not required because of the anonymized nature of patient records.

### 2.1 Study subjects and definitions

We retrieved data on consecutive women over 16 years of age with suspected NCCAH, referred for 250 µg CST from January 2001 – December 2020. As it is a real-life study, all CSTs for suspected NCCAH were included regardless of pretest 17OHP levels. The time of the CST was set as the index date. Subjects meeting inclusion criteria had clinical data available for at least 12 months before the index date.

Subjects using estrogen-containing oral contraceptives (OC), systemic or topical hormone replacement therapy (HRT), or systemic glucocorticoids (GC) were excluded (n=141). Subjects were considered as users of OC (n=109), HRT (n=3), or GC (n=29) if their prescription was filled within three months before the index date.

Clinical presentations that led to 17OHP measurements were hirsutism, oligomenorrhea, amenorrhea, acne, alopecia, or infertility that were identified within 12 months before the index date, based on the International Classification of Diseases-9 (ICD-9) or MHS internal diagnostic codes—called “Y” codes.

Demographic variables included age and last body mass index (BMI) at the index date. Laboratory values were defined as the last available value during the six months before or at the index date. Laboratory values included pre- and post-CST (basal and stimulated) serum 17OHP and cortisol, serum TSH, prolactin, total testosterone, dehydroepiandrosterone sulfate (DHEAS), androstenedione, LH, and FSH; LH to FSH ratio was also calculated.

NCCAH was defined as a 60-minute post-CST 17OHP serum level >30 nmol/L.

### 2.2 Biochemical analyses

Serum cortisol, TSH, prolactin, total testosterone, LH, and FSH levels were measured by chemiluminescent immunoassays (CLIAs) (Advia Centaur or Centaur XP, Siemens). Serum DHEAS levels were measured by CLIA (Immulite 2000, Siemens) from January 2000 through May 2014, and by CLIA (Centaur XP, Siemens) from June 2014 to December 2020. Serum androstenedione levels were measured using RIA (DSL) from January 2000 through December 2011, and by CLIA (Immulite 2000, Siemens) from January 2012 through December 2020.

From January 2000 through March 2015, serum 17OHP levels were assayed by direct RIA using Wizard gamma counter, Perkin-Elmer (OHP-CT: Cis Bio, Gif-sur-Yvette, France), and from April 2015 to December 2020 by ELISA (MG12181: Tecan IBL GmbH, Hamburg, Germany). The intra-assay coefficient of variation was 12.3-8.3% (range 0.345-13.21 nmol/L) for RIA, and 2.8-4.9% (range 7.39-34.57 nmol/L) for ELISA. The inter-assay coefficient of variation was 12-12.8% (range 2.94-22.81 nmol/L) for RIA, and 5.8-9.2% (range 0.78-17.39 nmol/L) for ELISA. The limit of detection was 0.09 nmol/L in both methods. MHS central laboratory performed validation for 17OHP assays, comparing 48 samples from their daily routine. Passing-Bablok regression revealed a good correlation between ELISA and RIA assays (r=0.97; range 0-25 nmol/L). The absolute 17OHP values measured by ELISA were slightly (34% on average) higher than by RIA (ELISA = 1.3462 RIA - 0.04); however, they were still within the allowable limits in accordance with the manufacturer’s package insert reference range.

### 2.3 Statistical analysis

Categorical variables were described as numbers and frequencies. Continuous variables were described as means and standard deviations (SD), or medians and ranges. The Chi-square test or Fisher’s exact test was used to compare categorical variables, and the student’s t-test was used to compare continuous variables, respectively. We allocated the individuals into two groups according to the 17OHP assay method used (RIA vs ELISA). For each group, a receiver-operating characteristic (ROC) curve was generated and an optimal basal 17OHP threshold with the highest sensitivity and specificity was determined. Positive (PPV) and negative (NPV) predictive values were calculated. We compared the sensitivity and specificity between the RIA and the ELISA groups when using the optimal RIA-based basal 17OHP cutoff. For each 17OHP assay group, univariate and multivariate logistic regression analyses were performed to identify variables that predict NCCAH, defined as a post-CST 17OHP serum level of >30 nmol/L. Variables with a p-value <0.1 on the univariate analysis were included in the multivariate analysis, which aimed to determine any independent predictors of NCCAH.

A two-sided P-value < 0.05 was considered statistically significant. All analyses were conducted with the statistical software SPSS, version 25 (IBM Corporation, Armonk, NY, USA).

## 3 Results

CST was performed in 2409 women who satisfied the inclusion criteria (1564 in the RIA group and 845 in the ELISA group). The mean (± SD) age was 24.1 ± 7 years. Symptoms that prompted cosyntropin testing were hirsutism in 45% of subjects, oligomenorrhea in 40%, amenorrhea in 25%, acne in 39.4%, infertility in 13.5%, and alopecia in 12.5%. The two groups were comparable in terms of the indication for testing, except for hirsutism which was more common in the RIA group (p<0.001) (**Table 1**).

**Table 1:** Clinical and biochemical characteristics of women in the RIA and the ELISA groups.

	RIA group	ELISA group	P-value
	(n=1564)	(n=845)	
Age, y			
Mean ± SD	24.1 ± 7.0	24.1 ± 7.4	0.83
Median (range)	22.4 (16–54)	22.2 (16-53.8)	
(n)	1564	845	
BMI, kg/m^2^			
Mean ± SD	25.5 ± 6.2	24.8 ± 5.6	0.52
Median (range)	24.2 (16.0-56.9)	23.6 (12.3-48.5)	
(n)	681	602	
Hirsutism			
n (%)	804 (51.4)	291 (34.4)	<0.001
Oligomenorrhea			
n (%)	629 (40.2)	335 (39.7)	0.83
Amenorrhea			
n (%)	393 (25.1)	211 (25)
Acne			
n (%)	615 (39.3)	336 (39.8)	0.87
Infertility			
n (%)	200 (12.8)	125 (14.8)	0.15
Alopecia			
n (%)	199 (12.7)	102 (12.1)	0.66
Basal 17OHP level*, nmol/L			
Mean ± SD	4.1 ± 6.4	5.9 ± 8.9	<0.001
Median (range)	2.6 (0.3-82.5)	3.6 (0.5-66.0)	
(n)	1564	845	
Stimulated 17OHP level, nmol/L			
Mean ± SD	9.9 ± 15.3	12.3 ± 17.3	<0.001
Median (range)	6.3 (0.4-82.5)	7.3 (0.5-66.0)	
(n)	1564	845	
NCCAH (stimulated 17OHP level>30nmol/L)			
n (%)	74 (4.7%)	63 (7.5%)	0.008

Basal cortisol level, nmol/L			
Mean ± SD	480.6 ± 196.6	430.4 ± 173.8	<0.001
Median (range)	453 (114.0-1487.0)	409 (67.0-1275.0)	
(n)	1365	765	
Stimulated cortisol level, nmol/L			
Mean ± SD	819.8 ± 209.8	807.2 ± 170.5	0.16
Median (range)	791 (143.0-2275.90)	795 (73.0-1594.0)	
(n)	1365	765	
Total testosterone, nmol/L			
Mean ± SD	2.2 ± 0.9	1.7 ± 0.7	<0.001
Median (range)	2.0 (0.6-9.70)	1.4 (0.4-8.6)	
(n)	1253	866	
Androstenedione, ng/ml			
Mean ± SD	2.9 ± 1.9	3.7 ± 1.8	<0.001
Median (range)	2.5 (0.3-16.9)	3.5 (0.3-11.0)	
(n)	1028	491	
DHEAS, µmol/L			
Mean ± SD	6.9 ± 3.6	6.8 ± 3.4	0.57
Median (range)	6.3 (0.4-22.8)	6.3 (0.4-19.5)	
(n)	1376	801	
LH, IU/L			
Mean ± SD	8.7 ± 7.9	8.7 ± 8.3	0.89
Median (range)	6.50 (0.5-76.9)	6.30 (0.5-68.0)	
(n)	1305	815	
FSH, IU/L			
Mean ± SD	5.5 ± 5.5	7.0 ± 7.4	<0.001
Median (range)	5.0 (0.7-111.8)	6.2 (0.7-116.7)	
(n)	1318	813	
LH to FSH ratio			
Mean ± SD	1.7 ± 1.5	1.4 ± 1.1	<0.001
(n)	1295	811	
Prolactin, mIU/L			
Mean ± SD	310.0 ± 247.4	280.5 ± 183.6	0.005
Median (range)	251.0 (18.0-3578.0)	228.0 (16.0-1999.0)	
	1198	731	
TSH, mIU/L			
Mean ± SD	2.4 ± 1.7	2.2 ± 1.4	0.013
Median (range)	2.1 (0.03-31.9)	2.0 (0.03-23.1)	
(n)	1412	845	

*For 17OHP, the intra-assay coefficient of variation was 9-22% for RIA and 13-25% for ELISA. The inter-assay coefficient of variation was 16%.

Continuous parameters are shown as mean ± SD and median (range), and categorical variables as n (%). 17OHP, 17-hydroxyprogesterone; BMI, body mass index; CST, cosyntropin stimulation test; DHEAS, dehydroepiandrosterone sulfate; FSH, follicle stimulating hormone; LH, luteinizing hormone; NCCAH, non-classic congenital adrenal hyperplasia; TSH, thyroid stimulating hormone.

The mean basal and stimulated 17OHP levels were lower in the RIA group as compared to the ELISA group (4.1 ± 6.4 vs. 5.9 ± 8.9 and 9.9 ± 15.3 vs. 12.3 ± 17.3, respectively, p<0.001 for both comparisons). Basal serum cortisol, total testosterone, and LH : FSH ratio were higher in the RIA group. Serum androstenedione levels were higher in the ELISA group (**Table 1**). The same pattern of differences was also observed between the two groups when comparing subjects without NCCAH (**Table 2**).

**Table 2:** Comparison of clinical and biochemical characteristics among women without NCCAH from the RIA and the ELISA groups.

	RIA group	ELISA group	P-value
	(n=1564)	(n=845)	
	Subjects without NCCAH	Subjects without NCCAH	
	(n=1492)	(n=782)	
Age, y			
Mean ± SD	24.0 ± 7.0	24.0 ± 7.3	1
Median (range)	22.3 (16–54)	22.0 (16-53.8)	
(n)	1492	782	
BMI, kg/m^2^			
Mean ± SD	25.4 ± 6.1	24.8 ± 5.6	0.08
Median (range)	24.1 (16.0-56.9)	23.6 (12.3-48.5)	
(n)	647	566	
Hirsutism			
n (%)	768 (51.5)	268 (34.3)	<0.001
Oligomenorrhea			
n (%)	614 (40.1)	314 (40.2)	0.67
Amenorrhea			
n (%)	376 (25.2)	201 (25.6)	0.83
Acne			
n (%)	590 (39.5)	309 (39.5)	0.99
Infertility			
n (%)	185 (12.4)	109 (13.9)	0.33
Alopecia			
n (%)	191 (12.8)	98 (12.5)	0.91
Basal 17OHP level*, nmol/L			
Mean ± SD	3.2 ± 2.5	4.0 ± 2.7	<0.001
Median (range)	2.5 (0.3-20.1)	3.4 (0.5-23.6)	
(n)	1492	782	
Stimulated 17OHP level, nmol/L			
Mean ± SD	6.8 ± 3.4	7.9 ± 3.9	<0.001
Median (range)	6.1 (0.4-29.3)	7.0 (0.5-29.1)	
(n)	1492	782	
Basal cortisol level, nmol/L			
Mean ± SD	480.7 ± 197.4	432.4 ± 172.3	<0.001
Median (range)	453 (114.0-1487.0)	408 (96.0-1275.0)	
(n)	1302	710	
Stimulated cortisol level, nmol/L			
Mean ± SD	829.4 ± 206.6	821.6 ± 162.4	0.39
Median (range)	796.0 (196.0-2275.9)	803.5 (240.0-1594.0)	
(n)	1302	710	
Total testosterone, nmol/L			
Mean ± SD	2.2 ± 0.9	1.6 ± 0.6	<0.001
Median (range)	2.0 (0.6-9.7)	1.4 (0.4-8.6)	
(n)	1194	807	
Androstenedione, ng/ml			
Mean ± SD	2.9 ± 1.9	3.6 ± 1.7	<0.001
Median (range)	2.5 (0.3-16.9)	3.45 (0.3-11.0)	
(n)	985	458	
DHEAS, µmol/L			
Mean ± SD	6.9 ± 3.6	6.8 ± 3.4	0.49
Median (range)	6.3 (0.4-22.8)	6.2 (0.4-19.5)	
(n)	1322	747	
LH, IU/L			
Mean ± SD	8.8 ± 8.0	8.6 ± 7.7	0.62
Median (range)	6.6 (0.5-76.9)	6.3 (0.5-68.0)	
(n)	1244	763	
FSH, IU/L			
Mean ± SD	5.4 ± 5.2	6.8 ± 5.7	<0.001
Median (range)	4.9 (0.7-111.8)	6.1 (0.7-104.0)	
(n)	1256	761	
LH to FSH ratio			
Mean ± SD	1.8 ± 1.6	1.4 ± 1.1	<0.001
(n)	1234	759	
Prolactin, mIU/L			
Mean ± SD	312.0 ± 251.4	281.3 ± 186.0	0.006
Median (range)	251.0 (18.0-3578.0)	228.0 (16.0-1999.0)	
(n)	1143	687	
TSH, mIU/L			
Mean ± SD	2.4 ± 1.7	2.2 ± 1.4	0.02
Median (range)	2.1 (0.03-31.9)	2.0 (0.03-23.1)	
(n)	1347	814	

*For 17OHP, the intra-assay coefficient of variation was 9% for RIA and 13% for ELISA. The inter-assay coefficient of variation was 18.5%.

Continuous parameters are shown as mean ± SD and median (range), and categorical variables as n (%). 17OHP, 17-hydroxyprogesterone; BMI, body mass index; CST, cosyntropin stimulation test; DHEAS, dehydroepiandrosterone sulfate; FSH, follicle stimulating hormone; LH, luteinizing hormone; NCCAH, non-classic congenital adrenal hyperplasia; TSH, thyroid stimulating hormone.

NCCAH was diagnosed in 74 (4.7%) subjects in the RIA group and 63 (7.5%) in the ELISA group (p=0.008) (**Table 1**). NCCAH subjects in the two groups had comparable parameters, except for higher total testosterone levels in the RIA group and higher androstenedione levels in the ELISA group (**Table 3**).

**Table 3:** Comparison of clinical and biochemical characteristics among women with NCCAH from the RIA and the ELISA groups.

	RIA group	ELISA group	P-value
	(n=1564)	(n=845)	
	Subjects with NCCAH	Subjects with NCCAH	
	(n=74)	(n=63)	
Age, y			
Mean ± SD	26.5 ± 8.1	26.1 ± 8.5	0.78
Median (range)	25.6 (16-52.7)	24.4 (16-50.3)	
(n)	74	63	
BMI, kg/m^2^			
Mean ± SD	26.2 ± 7.5	25.4 ± 5.6	0.61
Median (range)	25.1 (17.2-47.8)	24.9 (17-42.3)	
(n)	34	36	
Hirsutism			
n (%)	36 (48.6)	23 (36.5)	0.21
Oligomenorrhea			
n (%)	15 (20.3)	21 (33.3)	0.12
Amenorrhea			
n (%)	17 (23.0)	10 (15.9)	0.41
Acne			
n (%)	25 (33.8)	27 (42.9)	0.36
Infertility			
n (%)	15 (20.3)	16 (25.4)	0.26
Alopecia			
n (%)	8 (10.8)	4 (6.3)	0.54
Basal 17OHP level*, nmol/L			
Mean ± SD	23.5 ± 18.8	29.2 ± 20.2	0.09
Median (range)	16.6 (4.1-82.5)	20.6 (2.1-66.0)	
(n)	74	63	
Stimulated 17OHP level, nmol/L			
Mean ± SD	67.1 ± 16.1	63.5 ± 7.8	0.21
Median (range)	71.6 (30.9-82.5)	66.0 (32.8-66.0)	
(n)	74	63	
Basal cortisol level, nmol/L			
Mean ± SD	477.9 ± 179.6	434.3 ± 163.7	0.17
Median (range)	459.0 (155–949)	459 (67.0-948)	
(n)	63	55	
Stimulated cortisol level, nmol/L			
Mean ± SD	634.5 ± 160.5	641.5 ± 158.1	0.81
Median (range)	606.0 (143.0-1083.0)	637.0 (73.0-1092.0)	
(n)	63	55	
Total testosterone, nmol/L			
Mean ± SD	2.9 ± 0.9	2.0 ± 0.9	<0.001
Median (range)	2.9 (1.4-5.4)	1.7 (0.6-5.0)	
(n)	59	59	
Androstenedione, ng/ml			
Mean ± SD	4.2 ± 2.6	5.6 ± 2.4	0.04
Median (range)	3.3 (0.6-11.0)	5.9 (1.3-11.0)	
(n)	43	33	
DHEAS, µmol/L			
Mean ± SD	7.3 ± 3.5	7.3 ± 3.5	0.96
Median (range)	7.0 (1.2-18.2)	6.9 (0.7-16.9)	
(n)	54	54	
LH, IU/L			
Mean ± SD	5.9 ± 5.7	8.3 ± 8.2	0.08
Median (range)	4.2 (0.5-31.4)	5.6 (0.5-50.1)	
(n)	61	52	
FSH, IU/L			
Mean ± SD	6.7 ± 9.0	11.0 ± 18.9	0.12
Median (range)	5.6 (0.7-74.0)	6.30 (1.1-116.7)	
(n)	62	52	
LH to FSH ratio			
Mean ± SD	1.0 ± 0.7	1.1 ± 0.9	0.39
(n)	61	52	
Prolactin, mIU/L			
Mean ± SD	265.9 ± 134.6	268.1 ± 141.0	0.94
Median (range)	252.0 (58.0-584.0)	226.0 (119.0-792.0)	
(n)	55	44	
TSH, mIU/L			
Mean ± SD	2.4 ± 1.3	2.6 ± 1.6	0.97
Median (range)	2.1 (0.5-6.9)	2.0 (0.7-11.2)	
(n)	65	57	

*For 17OHP, the intra-assay coefficient of variation was 22% for RIA and 25% for ELISA. The inter-assay coefficient of variation was 13.5%.

Continuous parameters are shown as mean ± SD and median (range), and categorical variables as n (%).

17OHP, 17-hydroxyprogesterone; BMI, body mass index; CST, cosyntropin stimulation test; DHEAS, dehydroepiandrosterone sulfate; FSH, follicle stimulating hormone; LH, luteinizing hormone; NCCAH, non-classic congenital adrenal hyperplasia; TSH, thyroid stimulating hormone.

**Figure 1:**
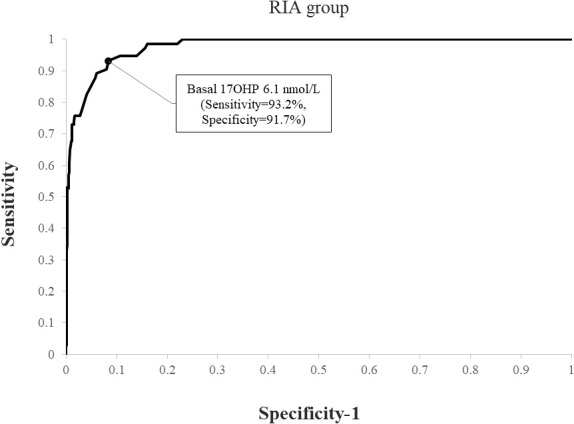
ROC curve analysis for determination of optimal basal 17OHP threshold in RIA (using a post-CST 17OHP cutoff > 30 nmol/L for the diagnosis of NCCAH).

**Figure 2:**
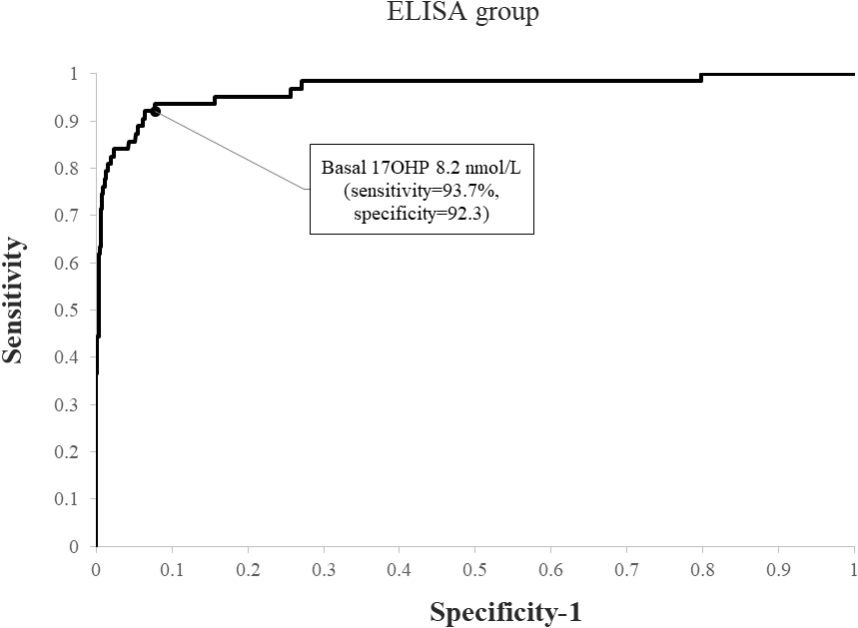
ROC curve analysis for determination of optimal basal 17OHP threshold in ELISA (using a post-CST 17OHP cutoff > 30 nmol/L for the diagnosis of NCCAH).

Using ROC analysis, the optimal basal 17OHP cutoff values predicting NCCAH were 6.1 nmol/L in the RIA group (sensitivity 93.2%, specificity 91.7%, NPV 99.6%, and PPV 35.8%) (**Figure 1**) and 8.2 nmol/L in the ELISA group (sensitivity 93.7%, specificity 92.3%, NPV 99.5%, PPV 49.6%) (**Figure 2**). When basal 17OHP cutoff value of 6.1 nmol/L was used in the ELISA group, sensitivity was 93.7% (p=1 for comparison with RIA), but specificity decreased to 84.5% (p<0.001 for comparison with RIA).

**Figure 3:**
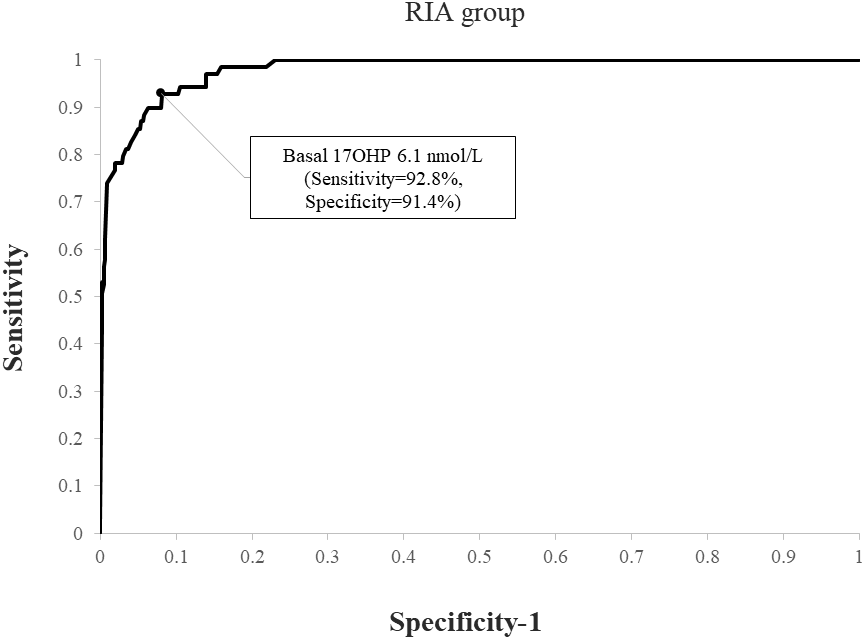
ROC curve analysis for determination of optimal basal 17OHP threshold in RIA (using a post-CST 17OHP cutoff > 40 nmol/L for the diagnosis of NCCAH).

**Figure 4:**
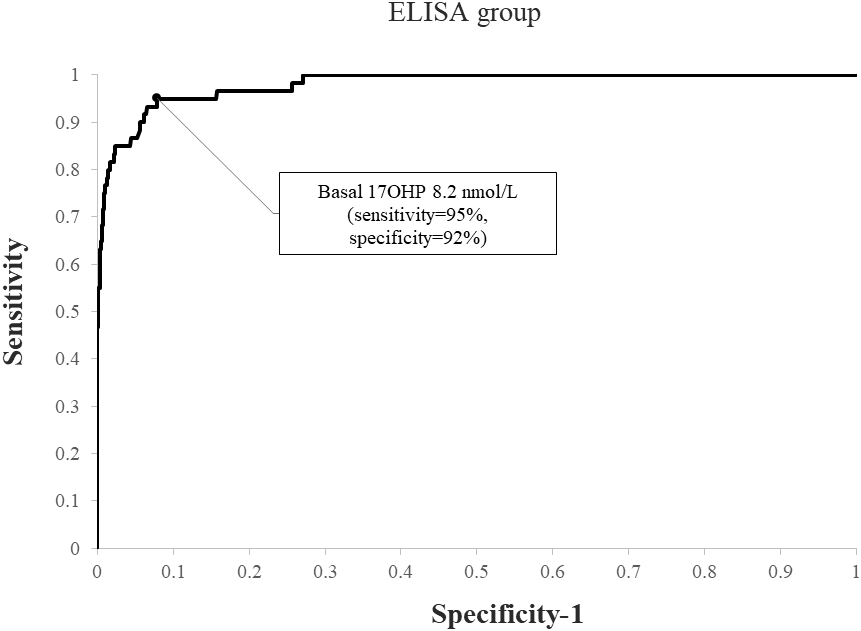
ROC curve analysis for determination of optimal basal 17OHP threshold in ELISA (using a post-CST 17OHP cutoff > 40 nmol/L for the diagnosis of NCCAH).

When applying a post-CST 17OHP diagnostic cutoff of > 40 nmol/L, NCCAH was diagnosed in 69 (4.4%) subjects in the RIA group and 60 (7.1%) in the ELISA group (p=0.007). Using ROC analysis, the optimal basal 17OHP cutoff values predicting NCCAH were 6.1 nmol/L in the RIA group (sensitivity 92.8%, specificity 91.4%, NPV 99.6%, PPV 33.2%) (**Figure 3**) and 8.2 nmol/L in the ELISA group (sensitivity 95%, specificity 92%, NPV 99.6%, PPV 47.5%) (**Figure 4**).

**Table 4** compares the clinical and biochemical characteristics of women with and without NCCAH (defined as a post-CST 17OHP serum level of >30 nmol/L) in the RIA and the ELISA groups. In both groups, the following variables met the criteria for inclusion in the multivariate logistic regression analysis: age, basal serum 17OHP, total testosterone, androstenedione, and LH : FSH ratio. A history of oligomenorrhea and a history of infertility were included in the analyses of the RIA and the ELISA groups, respectively.

**Table 4:** Comparison of clinical and biochemical characteristics between women with and without NCCAH in the RIA and the ELISA groups.

	RIA group		P-value	ELISA group		P-value
	(n=1564)			(n=845)		
	Subjects without NCCAH	Subjects with NCCAH		Subjects without NCCAH	Subjects with NCCAH	
	(n=1492)	(n=74)		(n=782)	(n=63)	
Age, y						
Mean ± SD	24.0 ± 7.0	26.5 ± 8.1	0.002	24.0 ± 7.3	26.1 ± 8.5	0.03
Median (range)	22.3 (16–54)	25.6 (16-52.7)		22.0 (16-53.8)	24.4 (16-50.3)	
(n)	1492	74		782	63	
BMI, kg/m^2^						
Mean ± SD	25.4 ± 6.1	26.2 ± 7.5	0.49	24.8 ± 5.6	25.4 ± 5.6	0.57
Median (range)	24.1 (16.0-56.9)	25.1 (17.2-47.8)		23.6 (12.3-48.5)	24.9 (17-42.3)	
(n)	647	34		566	36	
Hirsutism						
n (%)	768 (51.5)	36 (48.6)	0.72	268 (34.3)	23 (36.5)	0.82
Oligomenorrhea						
n (%)	614 (40.1)	15 (20.3)	<0.001	314 (40.2)	21 (33.3)	0.35
Amenorrhea						
n (%)	376 (25.2)	17 (23.0)	0.79	201 (25.6)	10 (15.9)	0.11
Acne						
n (%)	590 (39.5)	25 (33.8)	0.39	309 (39.5)	27 (42.9)	0.69
Infertility						
n (%)	185 (12.4)	15 (20.3)	0.07	109 (13.9)	16 (25.4)	0.02
Alopecia						
n (%)	191 (12.8)	8 (10.8)	0.69	98 (12.5)	4 (6.3)	0.21
Basal 17OHP level, nmol/L						
Mean ± SD	3.2 ± 2.5	23.5 ± 18.8	<0.001	4.0 ± 2.7	29.2 ± 20.2	<0.001
Median (range)	2.5 (0.3-20.1)	16.6 (4.1-82.5)		3.4 (0.5-23.6)	20.6 (2.1-66.0)	
(n)	1492	74		782	63	
Stimulated 17OHP level, nmol/L						
Mean ± SD	6.8 ± 3.4	67.1 ± 16.1	<0.001	7.9 ± 3.9	63.5 ± 7.8	<0.001
Median (range)	6.1 (0.4-29.3)	71.6 (30.9-82.5)		7.0 (0.5-29.1)	66.0 (32.8-66.0)	
(n)	1492	74		782	63	
Basal cortisol level, nmol/L						
Mean ± SD	480.7 ± 197.4	477.8 ± 179.6	0.91	432.4 ± 172.3	434.3 ± 163.7	0.94
Median (range)	453 (114.0-1487.0)	459.0 (155–949)		408 (96.0-1275.0)	459 (67.0-948)	
(n)	1302	63		710	55	
Stimulated cortisol level, nmol/L						
Mean ± SD	829.4 ± 206.7	634.5 ± 160.5	<0.001	821.6 ± 162.4	641.6 ± 158.1	<0.001
Median (range)	796.0 (196.0-2275.90)	606.0 (143.0-1083.0)		803.5 (240.0-1594.0)	637.0 (73.0-1092.0)	
(n)	1302	63		710	55	
Total testosterone, nmol/L						
Mean ± SD	2.2 ± 0.9	2.9 ± 0.9	<0.001	1.6 ± 0.7	2.0 ± 0.9	<0.001
Median (range)	2.0 (0.6-9.7)	2.9 (1.4-5.4)		1.35 (0.4-8.6)	1.7 (0.6-5.0)	
(n)	1194	59		807	59	
Androstenedione, ng/ml						
Mean ± SD	2.9 ± 1.9	4.2 ± 2.6	<0.001	3.6 ± 1.7	5.5 ± 2.5	<0.001
Median (range)	2.5 (0.3-16.9)	3.3 (0.6-11.0)		3.45 (0.3-11.0)	5.9 (1.3-11.0)	
(n)	985	43		458	33	
DHEAS, µmol/L						
Mean ± SD	6.9 ± 3.6	7.3 ± 3.5	0.41	6.8 ± 3.4	7.3 ± 3.5	0.31
Median (range)	6.3 (0.3-22.8)	7.0 (1.2-18.2)		6.2 (0.4-19.5)	6.9 (0.7-16.9)	
(n)	1322	54		747	54	
LH, IU/L						
Mean ± SD	8.8 ± 8.0	5.9 ± 5.7	0.007	8.6 ± 7.7	8.3 ± 8.2	0.79
Median (range)	6.6 (0.5-76.9)	4.2 (0.5-31.4)		6.3 (0.5-68.0)	5.6 (0.5-50.1)	
(n)	1244	61		763	52	
FSH, IU/L						
Mean ± SD	5.4 ± 5.2	6.7 ± 9.0	0.07	6.8 ± 5.7	11.0 ± 18.9	<0.001
Median (range)	4.9 (0.7-111.8)	5.6 (0.7-74.0)		6.1 (0.7-104)	6.30 (1.1-116.7)	
(n)	1256	62		761	52	
LH to FSH ratio						
Mean ± SD	1.8 ± 1.6	1.0 ± 0.70	<0.001	1.4 ± 1.1	1.1 ± 0.9	0.09
(n)	1234	61		759	52	
Prolactin, mIU/L						
Mean ± SD	312.0 ± 251.4	265.9 ± 134.6	0.18	281.3 ± 186.0	268.1 ± 141.0	0.64
Median (range)	251.0 (18.0-3578.0)	252.0 (58.0-584.0)		228.0 (16.0-1999.0)	226.0 (119.0-792.0)	
(n)	1143	55		687	44	
TSH, mIU/L						
Mean ± SD	2.4 ± 1.7	2.4 ± 1.3	0.89	2.2 ± 1.4	2.5 ± 1.6	0.25
Median (range)	2.0 (0.03-31.88)	2.1 (0.5-6.9)		2.0 (0.03-23.1)	2.0 (0.7-11.2)	
(n)	1347	65		814	57	

Continuous parameters are shown as mean ± SD and median (range), and categorical variables as n (%).

17OHP, 17-hydroxyprogesterone; BMI, body mass index; CST, cosyntropin stimulation test; DHEAS, dehydroepiandrosterone sulfate; FSH, follicle stimulating hormone; LH, luteinizing hormone; NCCAH, non-classic congenital adrenal hyperplasia; TSH, thyroid stimulating hormone.

In the multivariate analysis, higher basal 17OHP level (p<0.001), lower LH: FSH ratio (p=0.005), and oligomenorrhea (p=0.021) emerged as independent predictors of NCCAH in the RIA group. Higher basal 17OHP (p<0.001), androstenedione (p=0.004), and total testosterone (p=0.012) levels were identified as independent predictors of NCCAH in the ELISA group. Of note, a lower LH : FSH ratio showed a similar trend in ELISA but did not reach significance (p=0.054). The results of the multivariate analyses are shown in **Tables 5** and **6**.

**Table 5:** Multivariate analysis for predictors of NCCAH among the RIA group.

Variable	Odds ratio	95% confidence interval	P-value
Age, y	1.02	0.96-1.08	0.61
History of oligomenorrhea	0.08	0.01-0.69	0.021
Basal 17OHP level*, nmol/L	1.45	1.29-1.63	<0.001
Total testosterone, nmol/L	1.09	0.72-1.63	0.69
Androstenedione, ng/ml	1.06	0.82-1.38	0.65
LH to FSH ratio	0.22	0.08-0.63	0.005

*For 17OHP, the intra-assay coefficient of variation was 9-22% for RIA. The inter-assay coefficient of variation was 16%.

17OHP, 17-hydroxyprogesterone; CST, cosyntropin stimulation test; FSH, follicle stimulating hormone; LH, luteinizing hormone.

**Table 6:** Multivariate analysis for predictors of NCCAH among the ELISA group.

Variable	Odds ratio	95% confidence interval	P-value
Age, y	0.95	0.82-1.10	0.53
History of infertility	3.46	0.36-33.57	0.29
Basal 17OHP level*, nmol/L	1.74	1.41-2.14	<0.001
Total testosterone, nmol/L	2.41	1.21-4.79	0.012
Androstenedione, ng/ml	1.78	1.21-2.62	0.004
LH to FSH ratio	.38	0.14-1.01	0.054

*For 17OHP, the intra-assay coefficient of variation was 13-25% for ELISA. The inter-assay coefficient of variation was 16%.

17OHP, 17-hydroxyprogesterone; CST, cosyntropin stimulation test; FSH, follicle stimulating hormone; LH, luteinizing hormone.

Using ROC analysis, the optimal LH : FSH ratio cutoff values for predicting NCCAH were 1.4 in the RIA group (sensitivity 85.3%, specificity 46.8%, NPV 98.5%, PPV 7.3%) and 1.2 in the ELISA group (sensitivity 71.2%, specificity 43.4%, NPV 95.6%, PPV 7.9%).

In the RIA group, the combination of a serum basal 17OHP value below 6.1 and an LH : FSH ratio above 1.4 increased the NPV to 100%. In the ELISA group, the combination of a serum basal 17OHP value below 8.2 and LH : FSH ratio above 1.2 increased the NPV to 99.7%. Combining basal serum 17OHP values below the optimal cutoffs with an LH : FSH ratio > 2, yielded an increase in the NPV to 100% in both groups.

## 4 Discussion

This study showed that the optimal RIA-based basal 17OHP cutoff at which to refer a woman suspected of NCCAH to CST was 6.1 nmol/L, comparable with that recommended in the guidelines (6 nmol/L) (1). When using ELISA, we observed an upward shift in the basal 17OHP cutoff to 8.2 nmol/L. This result is in accordance with a recent report by Domagala et al. (15) who examined the validity of the currently accepted 17OHP threshold at which CST should be performed in 343 polish individuals with suspected NCCAH. They showed that the basal ELISA-based 17OHP value that best qualified patients for testing was 2.8 ng/mL (8.4 nmol/L), with a sensitivity and a specificity of 77.2% and 91.3%, respectively.

While LC-MS/MS-based 17OHP measurement has been recommended by the Endocrine Society for the screening of NCCAH due to 21-hydroxylase deficiency (1), the use of LC-MS/MS is expensive, labor consuming and therefore not widespread, especially in adult outpatient clinics. In addition, standard cutoffs for this technique are still evolving (10).

Immunoassays are still the assays most frequently used in clinical settings and present good performance in NCCAH diagnosis. The classical basal 17OHP cutoff value of 6 nmol/L for performing CST was established based on immunoassays, mainly RIA (1). This cutoff has been called into question by several studies, most of which used RIA assays (2). Maffazoli et al. showed that 6.2% of women with NCCAH could have been missed using the classical threshold and Bidet et al. reported that 8% of NCCAH women had basal 17OHP levels < 6 nmol/L (6, 7). Escobar-Morreale et al. suggested a lower basal 17OHP level (5.1 nmol/L) to improve the screening sensitivity for NCCAH diagnosis in a cohort of women with hyperandrogenism (9).

Nonetheless, in light of the widespread use of immunoassays and the replacement of RIA by the ELISA 17OHP assay, it is pertinent to check whether the guideline-recommended basal 17OHP cutoff applies. This is the first sizable study gleaning data from a large cohort of women with hyperandrogenism and comparing data between the old RIA and the new ELISA method.

In the present study, 137/2409 (5.7%) women were diagnosed with NCCAH, defined as a stimulated 17OHP level >30 nmol/L. This figure is similar to previous studies of hyperandrogenic females (16, 17). This implies a significant number of unnecessary and costly CSTs in a real-world setting. Thus, adopting a higher 17OHP cutoff in ELISA assays might reduce the number of unnecessary tests.

Noteworthy, NCCAH was diagnosed more often in the ELISA (7.5%) than the RIA (4.7%) group. This difference could be explained by the upward shift in 17OHP levels in the ELISA group (**Tables 1**, **2**) which might have led to more false-positive tests, although among subjects diagnosed with NCCAH the basal 17OHP levels did not differ significantly between assays (**Table 3**). This hypothesis is supported by the Ambroziak et al. study that used an ELISA-based 17OHP assay for the diagnosis of NCCAH among women with hyperandrogenism and observed a considerable probability of false-positive tests when based only on a stimulated 17OHP cutoff value of ≥30 nmol/l (18). They showed that among 21 women with pre- or post-CST 17OHP ≥30 nmol/l, NCCAH was confirmed by genetic testing only in five women, of whom four subjects were heterozygous carriers. They also showed that post-CST ELISA-based 17OHP <30 nmol/l excludes NCCAH with a high degree of confidence (18). In addition, Azziz et al. showed that when post-CST 17OHP values were within the range of 30–45 nmol/l, most patients were heterozygous carriers (19). In the absence of genetic data, we cannot rule out the possibility of more heterozygous carriers (of CYP21A2 monoallelic mutations) in the ELISA group.

The validation study, performed by MHS central laboratory, established that when using ELISA, the absolute 17OHP values are higher (34% on average) than that achieved if the same samples were analyzed using RIA. This raises a question of what effect would employing a 34% higher diagnostic 17OHP cutoff (~ 40 nmol/L) have on our results. Interestingly, applying a higher post-CST 17OHP cutoff of > 40 nmol/L did not affect the observed discordance in the diagnosis rates of NCCAH nor the optimal basal 17OHP thresholds in the RIA and the ELISA groups.

NCCAH diagnosis might be truly more prevalent in the ELISA group. This might be explained by the introduction of guidelines in recent years that led to more precise referrals and testing. The higher androstenedione levels in the ELISA group may remark a higher degree of clinical suspicion that led to more positive CSTs. However, further studies that incorporate genetic data are needed.

We observed that androstenedione levels were higher in the ELISA than the RIA group, while testosterone levels were higher in the RIA. We do not have a clear explanation for these differences; however, as the androstenedione assay was changed in 2012, assays were not identical in the two groups. Consequently, the difference in assays could explain these discrepancies, and the fact that we still have these differences between RIA and ELISA groups in both women with and without NCCAH (**Tables 2**, **3**), supports this assumption. Basal cortisol, TSH and prolactin levels were higher in the RIA than in the ELISA group. However, they did not differ in NCCAH subjects between the two groups (**Table 3**) and did not differ within each group of RIA and ELISA, between women with and without NCCAH (**Table 4**). We do not have a decent explanation for that. However, as it is a real-life and not a randomized controlled trial, we cannot exclude the possibility that other unknown factors may have played a role in these differences.

Remarkably, in the RIA group, lower LH: FSH ratio and oligomenorrhea emerged as independent predictors of NCCAH. In the ELISA group, higher androstenedione and total testosterone levels were independent predictors, while lower LH: FSH ratio showed a marginal trend of significance.

Polycystic ovary syndrome (PCOS) is an important differential diagnosis of NCCAH in women and is much more frequent (20). NCCAH and PCOS share several clinical and biochemical features. Basal 17OHP, androstenedione, and testosterone levels might be increased in both NCCAH and PCOS female patients (6). Thus, it is imperative to investigate new biochemical markers rather than 17OHP alone, to better differentiate between these entities. Although elevated LH : FSH ratio (usually >2) is much more prevalent in polycystic ovary syndrome (PCOS) than in NCCAH (21), its discriminatory utility has not been thoroughly addressed.

In our study, a low LH: FSH ratio emerged as an independent predictor of NCCAH in the RIA group and showed a similar trend in the ELISA group. We found that combining basal serum 17OHP below the suggested cutoff with an LH: FSH ratio > 2 increases the NPV for the diagnosis of NCCAH to 100% in both RIA and ELISA groups. To our knowledge, this is the first study to show that the LH : FSH ratio improves the NPV for the diagnosis of NCCAH.

Our study’s main strength is the large number of subjects in each group, which enabled several analyses and allowed drawing important implications for clinical practice. We studied females older than 16 years to avoid 17OHP level differences derived from different pubertal development (22).

This study has some limitations which stem from its retrospective design. An unknown confounding may have influenced our results. The results of this study may not be generalizable to users of other assays. We cannot rule out the inclusion of peri- or post-menopausal women in the study population that may have affected the results, although the median age was 22 years. The lack of genetic profiling is another key limitation, as it may have helped better define the NCCAH populations and draw further distinctions between the 17OHP-assay groups. Further studies that incorporate genetic data are mandatory.

In conclusion, our results suggest adopting a higher ELISA-based 17OHP cutoff (8.2 nmol/L) at which patients suspected of NCCAH should be referred for further evaluation. This may help eliminate the number of unnecessary tests. When using RIA, the classical 17OHP cutoff level of 6 nmol/L seems to apply. Utilization of LH : FSH ratio can improve the negative predictive value of the basal 17OHP levels.

## Data availability statement

The raw data supporting the conclusions of this article will be made available by the authors, without undue reservation.

## Ethics statement

The studies involving human participants were reviewed and approved by Maccabi Healthcare Services (MHS) institutional review board (IRB) and ethics committee. Written informed consent from the participants’ legal guardian/next of kin was not required to participate in this study in accordance with the national legislation and the institutional requirements.

## Author contributions

AN, LS, and MZ were responsible for the design of the study and data acquisition. AN, LS, and MZ contributed to the analysis and the interpretation of data, drafted the manuscript and revised it critically for important intellectual content. NS, NW, and MS-A contributed to the analysis and the interpretation of data and revised the manuscript critically for important intellectual content. LS-R, SA, and RG contributed to the interpretation of data. All authors read and approved the final manuscript.
